# Prognostic Significance of Serum Vitamin D Levels in Egyptian Females with Breast Cancer

**DOI:** 10.31557/APJCP.2019.20.4.983

**Published:** 2019

**Authors:** Hikmat Abdel-Razeq

**Affiliations:** *Departments of Internal Medicine, Section of Hematology and Medical Oncology, King Hussein Cancer Center and School of Medicine, University of Jordan, Amman, Jordan.*

**Dear Editor**

I read with great interest the paper published by Ismail et al., (2018) in your journal entitled “Prognostic significance of serum vitamin d levels in Egyptian females with breast cancer”. While I really thank the authors for addressing a very controversial and challenging topic, I have few points to address. The association, or the link, between vitamin-D deficiency and cancer in general, breast in particular, is old and controversial, at best! Several studies had linked Vitamin-D deficiency with increasing risk of having breast cancer (Crew et al., 2009), worse pathological features (Peppone et al., 2012) advanced stage at presentation and even poor treatment outcome (Chiba et al., 2017). Additionally, several studies had supported the vitamin D-cancer prevention hypothesis (Grant, 2018). However, many of such studies suffered methodology problems! Vitamin-D deficiency and breast cancer are both very common and such association may happen. Though many studies described a correlation between Vitamin-D concentration and disease stage; researchers questioned such association. Authors of one study (Jacobs et al., 2016) cited by the authors, concluded that “though their study confirmed previous work regarding the correlates of vitamin-D concentrations, it does not provide support for an association between vitamin D status and breast cancer stage”. In this study, 50 women with primary invasive, non-metastatic breast cancer were tested for vitamin-D level at diagnosis, before any cancer treatment. Fifteen (30.0%) patients were found to be vitamin-D deficient. All patients were followed up for a median of 30 months. It is hard to believe that a study that involved only 50 patients with only 15 of them had vitamin-D deficiency, recruited over a period of 4 years in one of the busiest national cancer centers with a median follow up of only 30 months can jump to such major conclusions. Reviewing the data presented in Table 2 of the current study, in a different way than presented, clearly shows major differences between both study groups (attached Table). Compared to those with normal vitamin-D level, deficient patients had larger tumor size (46.7% vs. 2.9%), more advanced-stage disease at presentation ( 53.3% vs. 2.9%), had tumors with higher grade (33.3% vs. 2.9%), hormone-receptor negative (73.3% vs. 51.4%) and HER-2 positive ( 86.7% vs. 40.0%). All these pathological features are well-established poor prognostic features and associated with poor disease-free and overall survival. It is also difficult to assume that low vitamin-D levels in a very small number of patients (n= 15) was the reason behind all such poor prognostic features. The multivariate analysis presented in the study showed that progesterone receptor status was the only factor independently affecting overall survival. Additionally, authors found out that stage II had worse survival compared to stage I with HR 4.8 (p = 0.042) while stage III compared to stage I had HR of 1.7 (p = 0.577). Such findings raised many questions about the validity of the final conclusions. As such, I believe that the conclusions made by the authors that vitamin D deficiency had a negative effect on overall and disease-free survival in their 15 vitamin-D deficient breast cancer patients is not supported and should not be stated based on this study.Given the huge amount of literature on this topic, I really doubt that a large prospective study can be carried out to further address this issue.

**Table A:** Pathological Features of Breast Cancer in Patient with Vitamin-D Deficient versus Those with No Deficiency

	Vitamin-D Deficiency		P-Value
Pathological Characteristics	Yes (n=15)	No (n=35)	
Tumor size ≥5 cm	7 (46.7%)	1 (2.9%)	0.0004
Grade-3	5 (33.3%)	1 (2.9%)	0.0069
Stage III	8 (53.3%)	1 (2.9%)	0.0001
Lymph Node positive	13 (86.7%)	17 (48.6%)	0.0138
ER-Negative	11 (73.3%)	18 (51.4%)	0.2146
HER2-Positive	13 (86.7%)	14 (40.0%)	0.0044

ER, Estrogen Receptor; HER2, Human Epidermal Growth Factor Receptor
